# Stress and odorant receptor feedback during a critical period after hatching regulates olfactory sensory neuron differentiation in *Drosophila*

**DOI:** 10.1371/journal.pbio.3001101

**Published:** 2021-04-01

**Authors:** Shadi Jafari, Johan Henriksson, Hua Yan, Mattias Alenius

**Affiliations:** 1 Department of Biomedical and Clinical Sciences, Linköping University, Linköping, Sweden; 2 Department of Molecular Biology, Umeå University, Umeå, Sweden; 3 Molecular Infection Medicine Sweden, Umeå Centre for Microbial Research, Department of Molecular Biology, Umeå University, Umeå, Sweden; 4 Department of Biology, University of Florida, Gainesville, Florida, United States of America; ICM, FRANCE

## Abstract

Here, we reveal that the regulation of *Drosophila* odorant receptor (OR) expression during the pupal stage is permissive and imprecise. We found that directly after hatching an OR feedback mechanism both directs and refines OR expression. We demonstrate that, as in mice, *dLsd1* and *Su(var)3-9* balance heterochromatin formation to direct OR expression. We show that the expressed OR induces *dLsd1* and *Su(var)3-9* expression, linking OR level and possibly function to OR expression. OR expression refinement shows a restricted duration, suggesting that a gene regulatory critical period brings olfactory sensory neuron differentiation to an end. Consistent with a change in differentiation, stress during the critical period represses *dLsd1* and *Su(var)3-9* expression and makes the early permissive OR expression permanent. This induced permissive gene regulatory state makes OR expression resilient to stress later in life. Hence, during a critical period OR feedback, similar to in mouse OR selection, defines adult OR expression in *Drosophila*.

## Abstract 2

This study reveals that the regulation of odorant receptor expression during the Drosophila pupal stage is permissive and imprecise; olfactory sensory neuron activity directly after hatching both directs and refines odorant receptor expression. Hence, during a critical period, activity feedback defines adult odorant expression in Drosophila, as happens in mouse.

## Introduction

Olfactory sensory neurons (OSNs) in most vertebrates and insects are specified to express a single odorant receptor (OR) from a large repertoire of OR genes in the genome [1–4]. Two OR gene regulatory models have been described: the vertebrate probabilistic selection model and the invertebrate predetermined instructive model.

The vertebrate OR regulatory model depends on chromatin state changes—from a repressed state to an active state and back again to a general repressed state [5,6]. In mice, non-expressed OR genes are embedded in constitutive heterochromatin marked by histone H3 lysine 9 trimethylation (H3K9me3) [5,7]. According to a mathematical model of OR regulation, a yet-to-be-identified H3K9me3 demethylase sporadically opens the constitutive heterochromatin at a single OR locus and initiates expression [6,8]. *Lsd1* erases histone H3 lysine 9 dimethylation (H3K9me2), which further opens the chromatin and establishes OR expression [6]. The expressed OR then induces several feedback loops that downregulate *Lsd1* and induce heterochromatin formation, blocking the additional initiation of OR expression [6,9–11]. Unknown transcription factors (TFs) restrict the expression of each mouse OR to a stereotyped region in the olfactory epithelium [12].

*Drosophila* OR expression is generally viewed as a developmentally predetermined and non-plastic process [13–15]. There are several reasons for this assumption. OR expression is stereotypically organized [1], and *Drosophila* OSNs are specified in a lineage-dependent manner [13]. Notch signaling splits OSNs into 2 subgroups with defined projection patterns and OR expression [16]. Defined TF combinations both drive and restrict OR expression [15,17–19].

Nevertheless, the odor environment and odor exposure early in life can modulate *Drosophila* OR expression and odor responses [20–22]. Thermal stress and starvation induce plasticity in *Drosophila* adult OR expression [23]. H3K9me2, which marks OR promoters in vertebrates, also marks OR genes in *Drosophila* OSNs [19]. *G9a*, which produces H3K9me2, restricts OR expression in *Drosophila* [24]. *Su(var)3-9*, which produces H3K9me3 and induces constitutive heterochromatin, suppresses spurious OR expression [19,23]. The OR *cis* regulatory regions support cooperative TF interactions that oppose heterochromatin and limit stress-induced plasticity [23,25]. Thus, a heterochromatin-regulated OR expression plasticity that is in some ways similar to that found in vertebrates also seems to exist in *Drosophila*.

Here, we further address the role of heterochromatin in *Drosophila* OR regulation. We first demonstrate that OR gene regulation stringency increases after a restricted time of heightened plasticity and a stress-sensitive period of early fly development. We show that *dLsd1* and *Su(var)3-9* initiate and maintain OR expression stringency in *Drosophila*. The expressed OR regulates *dLsd1* and *Su(var)3-9* expression, creating a feedback loop that restricts and balances OR expression. Stress during this period inhibits the feedback loop and produces permanent changes in OR expression.

## Results

### *Drosophila* chemoreceptor expression matures during the first few days of adult life

**Fig 1:**
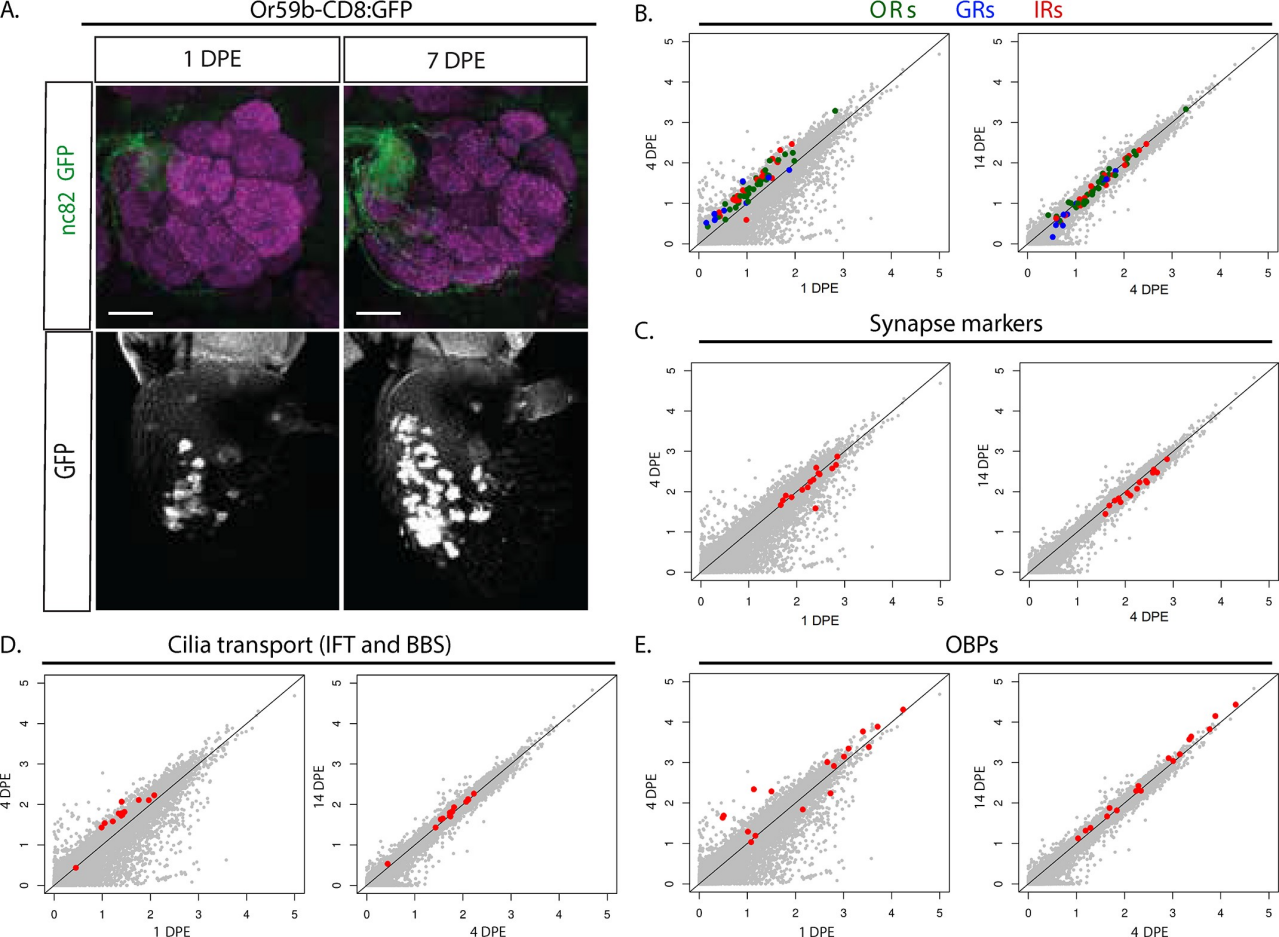
OR expression matures and OSN development continues after eclosion. (A) Whole-mount brain and antenna staining shows the *Or59b* reporter GFP expression (green) in 1- and 7-DPE flies. Synaptic neuropil regions are labeled with the presynaptic marker nc82 (magenta). Scale bar denotes 3.5 μm. Below each merged image, the GFP expression in the antenna is shown as the white channel. Note the increased expression and uniform level of expression between OSNs in the 7-day flies. (B–E) Degree of change in RNA sequencing read counts observed at 4 DPE relative to 1 DPE (left) and at 14 DPE relative to 4 DPE (right). Normalized logarithmic counts (log10 size-factor-normalized counts) for each gene from the respective sample were scatter-plotted. Data and statistics are in S1 Data. The raw sequencing data are available on ArrayExpress (#E-MTAB-9805). The code is available on Github (https://github.com/henriksson-lab/mattias-or). Genes shown in grey except (B) ORs (green), GRs (blue), and IRs (red); (C) synapse genes (red); (D) IFT and BBS genes (red); and (E) OBPs (red). The line is the reference at which gene expression is the same between conditions. BBS, BBSome; DPE, days post-eclosion; GR, gustatory receptor; IFT, intraflagellar transport; IR, ionotropic receptor; OBP, odorant binding protein; OR, odorant receptor; OSN, olfactory sensory neuron; RNA-seq, RNA sequencing; TF, transcription factor.

We and others have observed that OR reporter expression varies between OSNs in day-old flies, rising to the uniform high level observed in adult flies after a few days (Fig 1A) [26]. To further investigate OR expression dynamics, we performed RNA sequencing (RNA-seq) analyses comparing antennae from flies 1 day (newly hatched), 4 days (around the point of uniform OR expression), and 14 days (mature) post-eclosion. For each time point biological triplicates were analyzed. Strikingly, all 30 of the 34 adult antennal ORs as well as the olfactory co-receptor Orco increased significantly in expression between 1 and 4 days post-eclosion (DPE) (Fig 1B; S1 Data), but stopped increasing after day 4. The expression of the ionotropic receptors (IRs) and gustatory receptors (GRs) also increased during the first 4 DPE (Fig 1B; S1 Data). We found that 13 of 22 antennal IRs and 8 of 10 GRs expressed in OSNs increased 1-fold or more. As with the ORs, any changes in IR and GR expression after day 4 were minor, without any discernible pattern (Fig 1B; S1 Data). Thus, chemoreceptor expression in general seems to mature during the first 4 DPE.

During the same period, OSN connectivity is also maturing [26–28]. Analysis of the synaptic gene network showed a slight decrease but no uniform change in expression of the genes in the synaptic network from day 1 to day 4 (Fig 1C; S1 Data). This indicates that a limited set of genes or separate post-transcriptional mechanisms are responsible for refining OSN synapses. We next expanded the analysis further to include other OSN gene networks. Sensory neurons are the only ciliated cells in *Drosophila*, and the ciliary transport machinery (e.g., intraflagellar transport [IFT] and BBSome [BBS]) is important for the ciliary localization of the chemoreceptors [29]. In OSNs, olfactory transduction levels are affected by OR levels as more ORs are transported into the cilia [29]. Interestingly, we found increasing expression of the IFT and BBS genes during the first 4 DPE and no further change after the fourth day (Fig 1D; S1 Data). OSNs also express high levels of another auxiliary set of olfactory proteins required for specific odor responses, the odorant binding proteins (OBPs) [30]. The expression of most OBP genes either remains steady or increases from day 1 to day 4 (Fig 1E; S1 Data), lending further support to the idea that OSNs continue to develop and sensory transduction continues to change after the pupal stage.

### Stress modulates the maturation of OR expression

**Fig 2:**
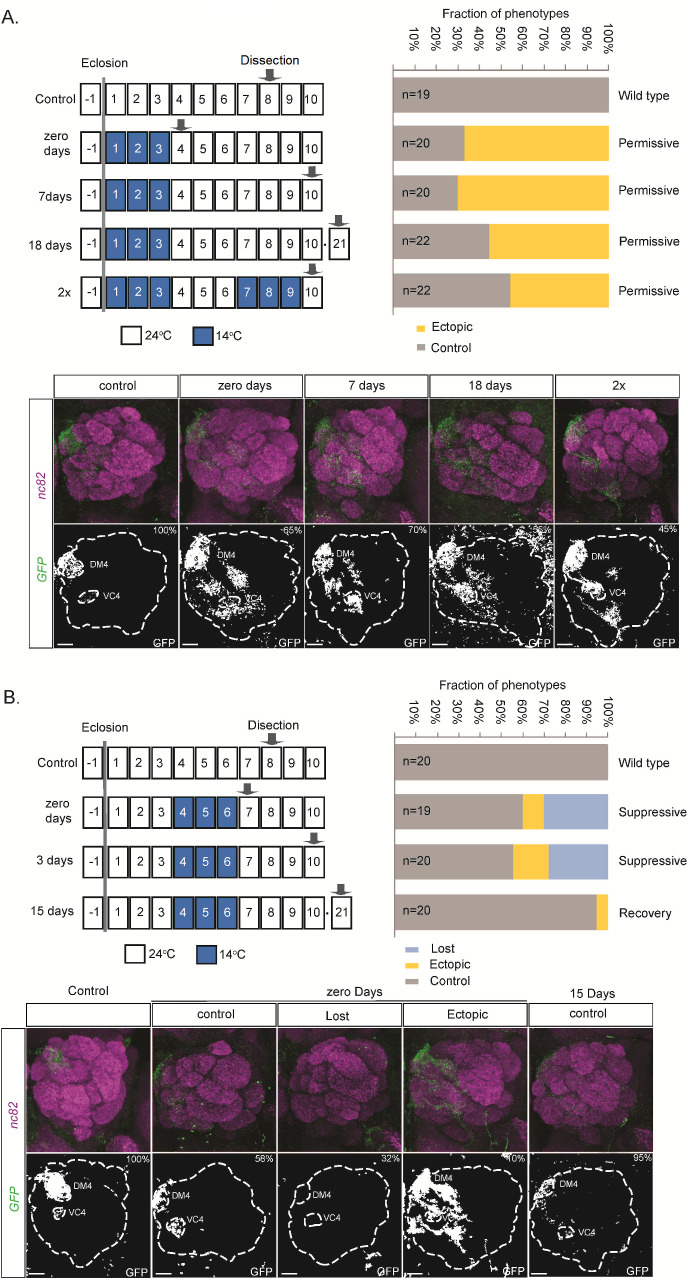
Permanent OR gene regulation changes following environmental stress during the OR expression maturation. The schematic drawing shows the time points of thermal stress treatments and sample preparation. The graphs show the fraction of flies with control, lost, or ectopic *Or59bME* expression after 3 days of thermal stress initiated on (A) day 1 or (B) day 4. The recovery time at ambient temperature is denoted in days (S2 Data). The antennal lobes represent the analyzed phenotypes. GFP expression (green) is driven by the *Or59b* minimal reporter. Synaptic neuropil regions are labeled with the presynaptic marker nc82 (magenta). The percentages show the fraction of the phenotype that is presented in that panel. Scale bar denotes 3.5 μm. Note the persistent ectopic expression after 18 days of recovery or after a second exposure to low temperature (2×). The loss phenotype reverted to single OSN class expression after 14 days of recovery at room temperature. OR, odorant receptor; OSN, olfactory sensory neuron.

We have previously observed that starvation and thermal stress increase OR expression plasticity [23] and that cooperative TF interactions in the *cis* regulatory region stabilize the OR expression. In these studies, we focused on adult (5–7 DPE) flies, but the stress-induced plasticity suggested that stress could change also the OR expression maturation process. To visualize stress-induced modulation of OR expression at all stages, we used the *Or59b minimal enhancer (Or59bME)*, an *Or59b* reporter that lacks the cooperative regulation region required to resist stress-induced changes [23]. After dissection and whole-mount staining of the brain, we analyzed the innervation of the antenna lobe. At room temperature (24°C), *Or59bME* behaves just like the endogenous *Or59b* gene, with its expression restricted to the ab2a OSN class [1,2,23] (Fig 2A). Reducing the temperature alters *Or59bME* reporter expression, but the timing of the temperature shift dictates the resulting phenotype (Fig 2). We found that shifts during the first 3 DPE led to stereotype ectopic *Or59bME* expression in several OSN classes, as evidenced by the appearance of multiple GFP-positive glomeruli in the antennal lobe (Fig 2A). But all temperature shifts after day 3 produced loss-of-expression phenotypes (Fig 2B). This sharp transition suggests a drastic change in OSN gene regulation. It also suggests stress may alter terminal OSN differentiation.

If stress modulates terminal OSN differentiation, the ectopic expression phenotypes we observed with early temperature shifts could be expected to become permanent. Indeed, when we returned *Or59bME* flies that underwent early temperature shifts to room temperature, we found that the stress-induced ectopic *Or59bME* reporter expression pattern persisted throughout a 7-day recovery period (Fig 2A). It even remained similar after a prolonged 18-day recovery period (Fig 2A). If the process of OR expression maturation is the final stage of OSN differentiation, then temperature shifts after maturation is complete should be reversible. Consistent with this hypothesis, we found that shifts back to room temperature for those exposed to thermal stress after day 3 led to a restoration of the expression pattern to a single OSN class (Fig 2B). This indicates that the OR expression state was already fixed when the flies were subjected to the temperature shift. To address this further, we subjected flies carrying *Or59bME* to 2 cold shifts, one during the critical period and another after a 3-day recovery period. As expected, the resulting *Or59bME* ectopic expression pattern for flies subjected to shifts was similar to that of flies subjected to a single early shift (Fig 2A). Together, these results indicate that stress during the maturation phase switches adult OR expression from a stress-sensitive, refined expression pattern to a potentially less refined but stress-resilient expression pattern.

### OR feedback refines OR expression

**Fig 3:**
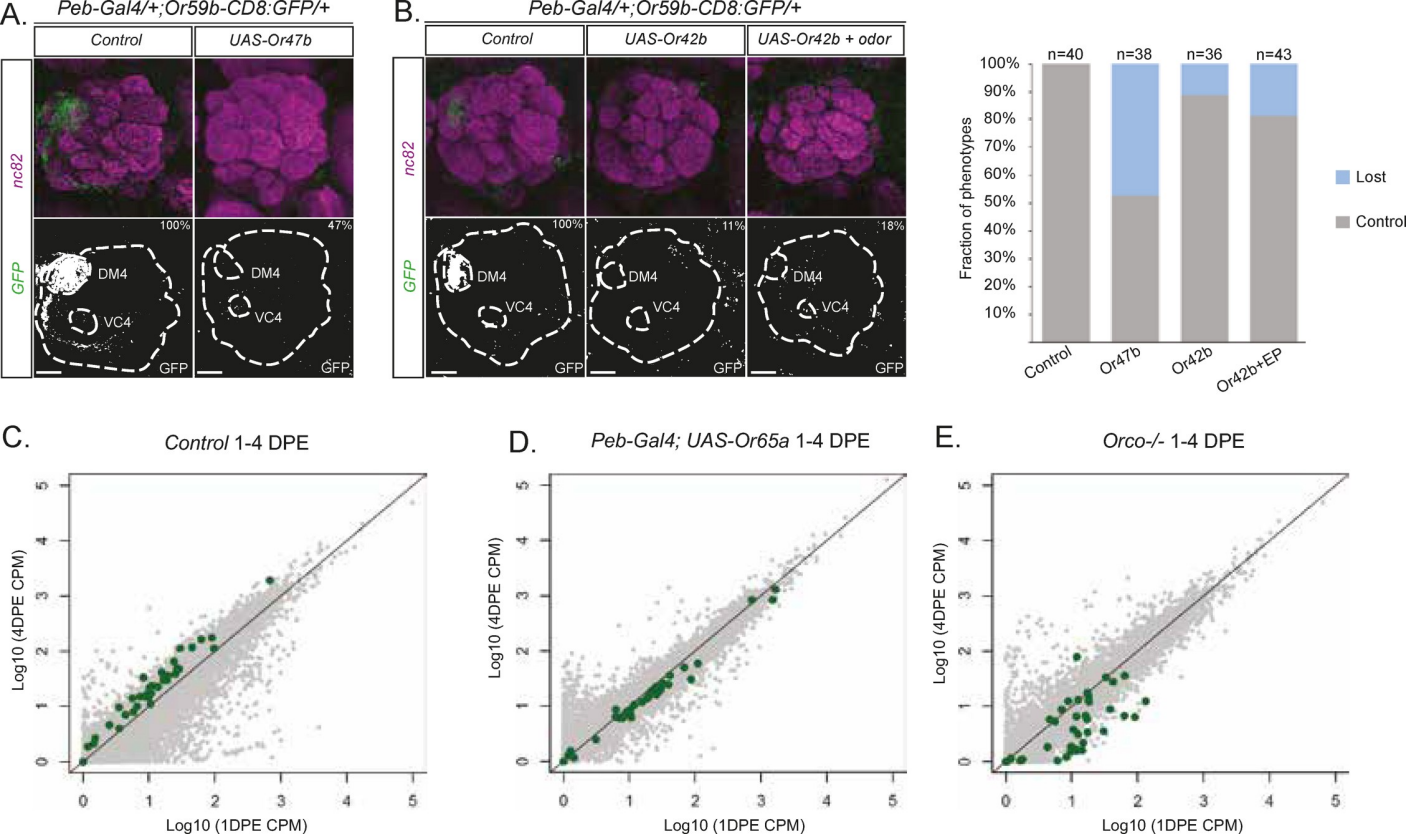
OR activity regulates OR expression. *Or59b* reporter GFP expression is shown in green, and synaptic neuropil regions are labeled with the presynaptic marker nc82 (magenta). Below each merged image, the GFP channel is shown. Antennal lobe and labeled glomeruli are marked. Control flies were crossed to *w*^*1118*^. Percentage denotes the proportion of flies with the depicted phenotype (S2 Data). Scale bar denotes 3.5 μm. Ectopic expression of *Or47b* (A) or *Or42b* (B) inhibits *Or59b* reporter expression. The loss of GFP expression is greater when flies with ectopic *Or42b* expression are exposed to the *Or42b*-specific odor ligand (EP). (C–E) Degree of change in RNA sequencing read counts observed between 1 and 4 DPE. Normalized logarithmic counts (log10 size-factor-normalized counts) for each gene from the respective sample were scatter-plotted. Genes shown in grey except ORs (green), for (C) control, (D) *Peb-Gal4;UAS-Or65a*, and (E) *Orco−/−*. Note that the increase in OR expression between day 1 and 4 shifts to suppression in olfactory sensory neurons with over-activity (D) and lost activity (E). The line is the reference at which gene expression is the same between conditions. Statistics for the figure are in S3 Data. The raw sequencing data are available on ArrayExpress (#E-MTAB-9805). The code is available on Github (https://github.com/henriksson-lab/mattias-or). CPM, counts per million; DPE, days post-eclosion; EP, ethyl propionate; OR, odorant receptor.

In mosquitoes, ectopic OR expression suppresses endogenous OR expression [31]. To determine whether OR expression level or function acts in a feedback mechanism on OR expression in *Drosophila* as well, we expressed an OR in all OSNs with *Peb-Gal4* and monitored *Or59b-CD8*:*GFP* reporter expression. With the exception of the male pheromone receptor Or47b, most *Drosophila* ORs have low spontaneous activity [32]. We found that about half of the flies with ectopic *Or47b* expression lost the *Or59b* reporter expression (Fig 3A and 3B), indicating that high spontaneous OR activity can suppress OR expression. Interestingly, ectopic expression of *Or42b*, an OR with lower spontaneous activity compared to *Or47b*, induced *Or59b* reporter expression loss in only 11% of the resulting flies (Fig 3A and 3B). To determine whether odor responses induce this negative feedback, we exposed flies to ethyl propionate (EP; diluted 10^−4^), a strong Or42b ligand. Flies with ectopic *Or42b* expression exposed to EP showed a slightly larger but still insignificant loss of *Or59b* reporter expression (11% versus 18%; Fig 3B). EP exposure of control flies (without ectopic *Or42b* expression) did not affect *Or59b* reporter expression (Fig 3B). Together, these results indicate OR expression level can feed back on and shape OR gene expression.

Next, we performed an RNA-seq experiment on antennae from flies with ectopic *Or65a* expression (Fig 3C and 3D). For 30 out of 34 antennal ORs, OR expression decreased in the flies with ectopic *Or65a* expression between 1 and 4 DPE (Fig 3D; S3 Data). Comparing OR expression with age-matched controls showed that the timing of the feedback regulation of OR expression depended on OSN lineage. In day-old (1 DPE) flies with ectopic *Or65a* expression, most trichoid-related ORs increased in expression (8/12; S3 Data; S1 Fig), whereas basiconic-related OR expression changes were minor. After OR expression maturation (4 DPE), basiconic-related OR expression was down-regulated, and the trichoid-related OR expression changes were less penetrant (S1 Fig; S2 Data), suggesting that OR feedback establishes trichoid-related OR expression during the pupal stage and restricts basiconic-related OR expression post-eclosion.

To further address whether OSN activity is required for OR expression, we performed an RNA-seq experiment on antennae from *Orco* mutant flies, in which most of the OSNs lack OR activity [33]. We found a drastic reduction in OR expression in *Orco* mutant flies 1–4 DPE (Fig 3E). This suggests Orco, and likely OR, function is important for this feedback regulation of OR expression. Consistent with our ectopic expression results in day-old *Orco* mutant flies, most trichoid-related ORs were up-regulated, while basiconic-related ORs showed no consistent directionality in the changes (S1 Fig). At 4 DPE, the expression of most trichoid and all basiconic ORs was reduced in the *Orco* mutant compared to controls, indicating that OR feedback post-eclosion is required in non-stress conditions to establish OR expression.

### The balance between *dLsd1* and *Su(var)3-9* refines OR expression

**Fig 4:**
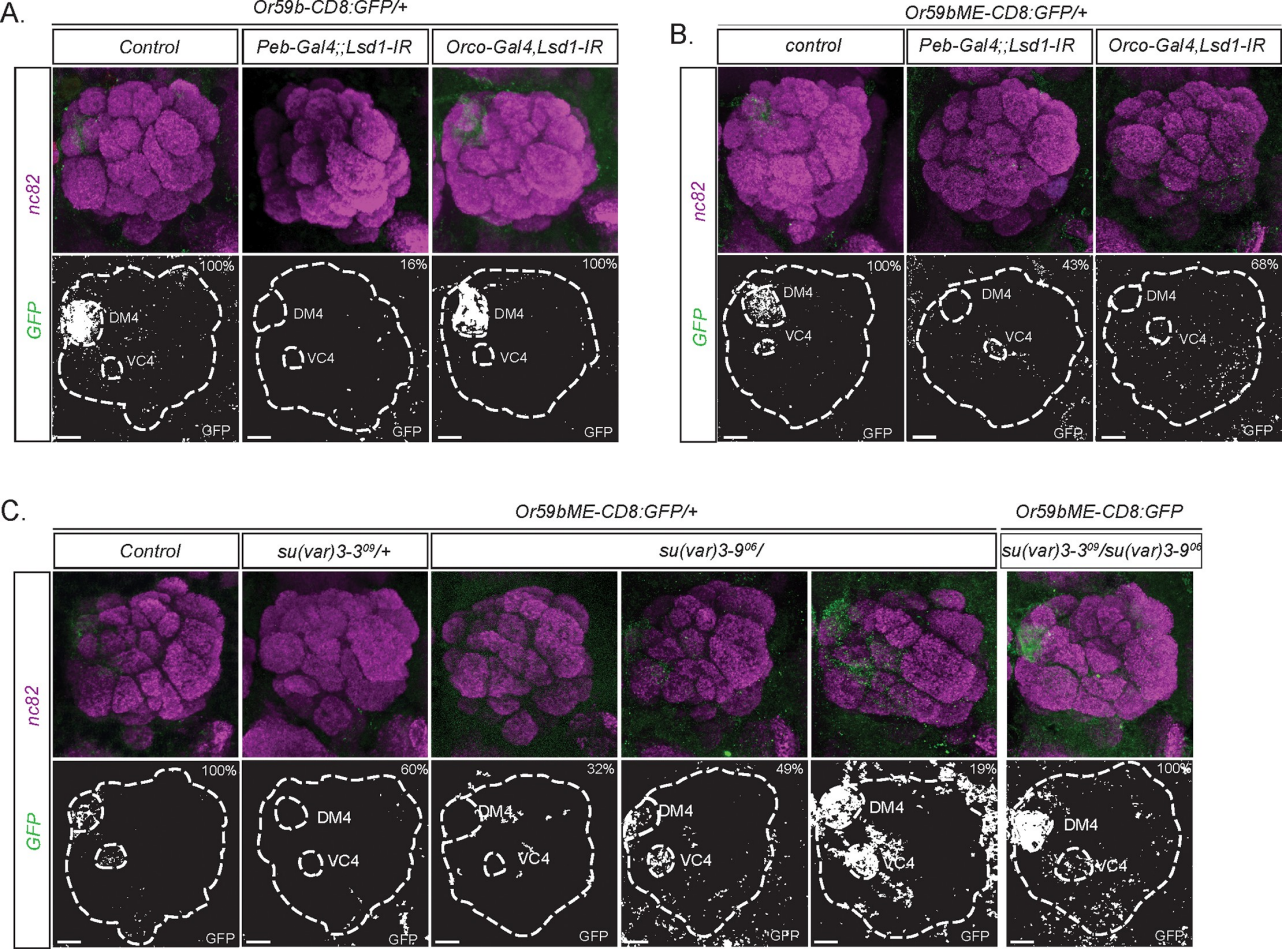
*dLsd1* balances *Su(var)3-9* and establishes *Or59b* expression. (A) *Or59b* reporter and (B) *Or59bME* reporter GFP expression in flies with *dLsd1* knock-down before initiation (*Peb-Gal4*) or after odorant receptor expression (*Orco-Gal4*). GFP expression is shown in green. Synaptic neuropil regions are labeled with the presynaptic marker nc82 (magenta). (C) *Or59bME* reporter expression in heterozygote *Su(var)3−9*^*06*^, heterozygote *dLsd1*^*09*^, and double heterozygote flies. Note that the expression changes of the *Or59bME* reporter in single heterozygote flies were rescued in *Su(var)3−9*^*06*^ and *dLsd1*^*09*^ heterozygote flies. Control flies were crossed to *w*^*1118*^. Percentage denotes the fraction of flies with the depicted phenotype (S2 Data). Scale bar denotes 3.5 μm.

The similarity of the OR feedback we observed to the vertebrate OR choice mechanism suggested a conserved OR regulatory mechanism. In mouse OSNs, Lsd1 catalyzes the demethylation of H3K9me2, opening heterochromatin to initiate OR expression [6]. To determine whether *dLsd1 (Su(var)3-3)* is also important for *Drosophila* OR expression, we used *Peb-Gal4* to express a UAS-IR (“IR” for “inverted repeats”) line specific to *dLsd1* in all OSNs. We found that 16% of the *dLsd1-*depleted flies showed loss of *Or59b* reporter expression (Fig 4A), suggesting that *dLsd1* is important in the establishment of OR expression in *Drosophila*. Interestingly, we found that many more *dLsd1*-depleted flies (43%) showed loss of the *Or59bME* reporter (Fig 4A and 4B), which lacks cooperative regulation, than loss of the *Or59b* reporter. This suggests TF cooperativity and *dLsd1* are both important for OR expression in flies. In mice, one of the few TFs known to regulate vertebrate OR expression, Lhx2 [34], requires cooperativity to maintain OR expression and counteract heterochromatin formation in OSNs [35].

During *Drosophila* development, *dLsd1* erases H3K4 dimethylation and promotes heterochromatin formation [36,37]. In both *Drosophila* and mice, *Su(var)3-9* methylates H3K9me2 to form H3K9me3, a marker of heterochromatin [38–40]. *Or59bME* reporter expression in heterozygous *Su(var)3-9* mutant flies shows a complex phenotype [23], with 19% of the flies showing ectopic expression, 32% showing loss of expression, and the rest showing single-class expression. *Or59bME* expression is also lost in 60% of heterozygous *Su(var)3−3*^*09*^ (*dLsd1* mutant) flies (Fig 4C). Combining the *dLsd1* and *Su(var)3-9* heterozygotes resets the balance and rescues reporter expression (Fig 4C). This suggests not only that the opening and closing of heterochromatin controls OR expression, but also that *dLsd1* promotes open heterochromatin in *Drosophila* OSNs to support OR expression.

To determine whether *dLsd1* initiates or maintains OR expression, we knocked down *dLsd1* using *Orco-Gal4*, which drives expression in most OSNs after OR expression has already begun [33,41]. In these late knock-down flies, *Or59b* reporter expression was unperturbed (Fig 4A), indicating *dLsd1* is required only during the initiation of OR expression. Interestingly, however, when we repeated the late *dLsd1* knock-down experiment with the *Or59bME* reporter, we found a strong loss-of-expression phenotype (Fig 4B), showing that *dLsd1* is required continuously to support OR expression.

### *Kdm4b* initiates OR expression

**Fig 5:**
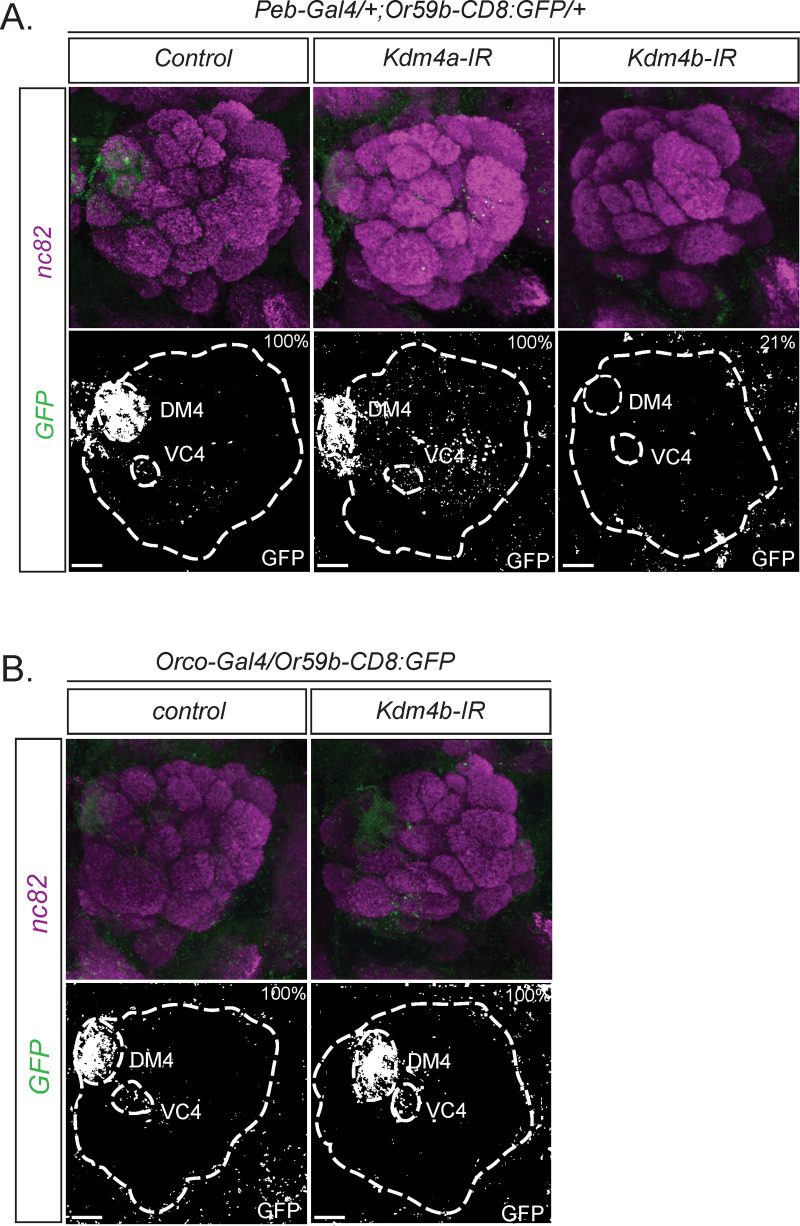
*Kdm4b* initiates *Or59b* expression. Whole-mount brain staining shows *Or59b* reporter expression of GFP (green). Synaptic neuropil regions are labeled with the presynaptic marker nc82 (magenta). Scale bar denotes 3.5 μm. (A) Loss of expression of the *Or59b* reporter is observed in the knock-down of *Kdm4b* but not *Kdm4a*. Control flies were crossed to *w*^*1118*^. (B) *Orco-Gal4* knock-down of *Kdm4b* after the initiation of odorant receptor expression. Percentage denotes the proportion of flies with the depicted phenotype. Control flies were crossed to *w*^*1118*^ (S2 Data).

Some mathematical models predict an as-yet-unknown factor that functions at individual OR loci in vertebrates to open constitutive heterochromatin by erasing H3K9me3 [6,8]. There are 2 genes encoding H3K9me3 demethylases in the *Drosophila* genome, *Kdm4a* (*Kdm4B* in vertebrates) and *Kdm4b* (*Kdm4A*, *-C*, *-D*, *-E* in vertebrates) [42]. We found, via knock-down of these 2 H3K9me3 demethylases in OSNs, that *Kdm4b* but not *Kdm4a* is required for *Or59b* expression (Fig 5A). *Kdm4b* is the major H3K9 demethylase in *Drosophila* [43], which is consistent with the hypothesis that the opening of heterochromatin is required for *Or59b* expression. We next asked whether *Kdm4b* is required for continuous *Or59b* expression by knocking down *Kdm4b* after OR initiation. Because OR expression begins in the mid-pupal stage and *Orco* expression begins shortly before eclosion, we decided to use *Orco-Gal4* [33] for this knock-down experiment. We found that *Orco-Gal4*-driven *Kdm4b* knock-down had no effect on *Or59b* expression (Fig 5B), indicating that *Kdm4b* is important for *Or59b* expression initiation rather than maintenance.

### OR feedback regulates *Kdm4b*, *dLsd1*, and *Su(var)3-9* expression

**Fig 6:**
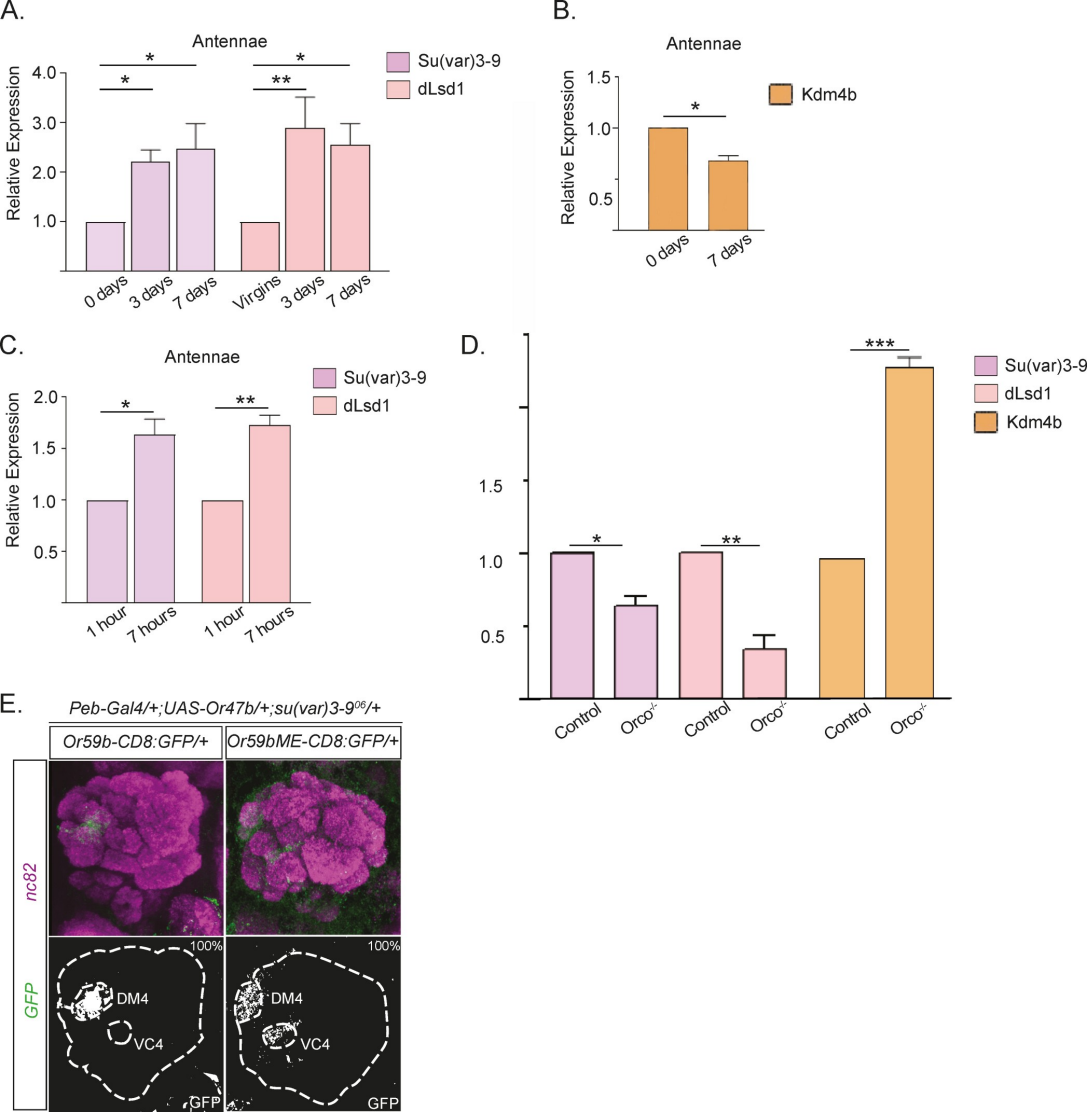
Dynamic expression of chromatin modulators regulates odorant receptor expression. (A) The graph shows *Su(var)3-9* and *dLsd1* mRNA levels in antenna at 1, 3, and 7 DPE (*p < 0.05; **p < 0.01; ***p < 0.001; error bars represent SEM (S2 Data). (B) The graph shows the *Kdm4b* mRNA levels in the antenna at 1 and 7days post-eclosion (DPE). Note that *Su(var)3-9*/*dLsd1* shows contrasting regulation to *Kdm4b* after eclosion. (C) This graph shows the mRNA levels of *Su(var)3-9* and *dLsd1* in the antenna at 1 hour and 7 hours after eclosion. Note that the expression levels increase to almost double at 7 hours post-eclosion. (D) The graph compares control (*w*^*1118*^) and *Orco* mutant mRNA levels of *Su(var)3-9*, *dLsd1*, and *Kdm4b* in the antenna at 4 DPE. Note that the expression levels are lower for *Su(var)3-9* and *dLsd1* and higher for *Kdm4b* in *Orco* mutant flies. (E) GFP expression (green) driven by the *Or59b* reporter. Note that the loss of *Or59b* reporter expression in flies with *Or47b* ectopic expression is rescued in a *Su(var)3−9*^06^ heterozygote background. Synaptic neuropil is labeled with the presynaptic marker nc82 (magenta). Control flies were crossed to *w*^*1118*^ (S4 Data).

Thus far, our results have revealed that the maturation of OR expression comprises a shift from a developmentally permissive state to a more restrictive state in adults. This suggests that expression levels of *dLsd1* and *Su(var)3-9* are dynamic. We therefore analyzed antennal expression of *Su(var)3-9* and *dLsd1* and found that expression increased from low levels in newly eclosed flies to adult levels 3 days later (Fig 6A). *Kdm4b* expression, in contrast, decreased over the same period (Fig 6B), suggesting that the maturation of OR expression involves a reduction in the initiation and manifestation of OR expression. A more detailed *dLsd1* and *Su(var)3-9* expression analysis showed that the main increase in mRNA levels for these 2 enzymes occurred during the first hours post-eclosion (Fig 6C). *Orco* mutant flies that lack OR activity also showed reduced *dLsd1* and *Su(var)3-9* mRNA levels (Fig 6D), suggesting that OR function or increased expression may induce *dLsd1* and *Su(var)3-9* expression. Interestingly, we found that *Kdm4b* mRNA levels also increased in Orco mutants 3 DPE compared to controls (Fig 6D). This suggests that the absence of OR expression and its feedback suppression of *Kdm4b* likely increases OR expression initiation. Next, we over-expressed *Or47b* in a heterozygous *Su(var)3-9* mutant background. We found, consistent with the hypothesis that increased OR expression or OR activity induces heterochromatin formation, that the heterozygote mutant reduction of *Su(var)3-9* balanced the effect of *Or47b* ectopic expression and rescued the loss of *Or59b* reporter expression (Fig 6E).

### Stress regulates *Su(var)3-9* expression differently during and after OR expression maturation

**Fig 7:**
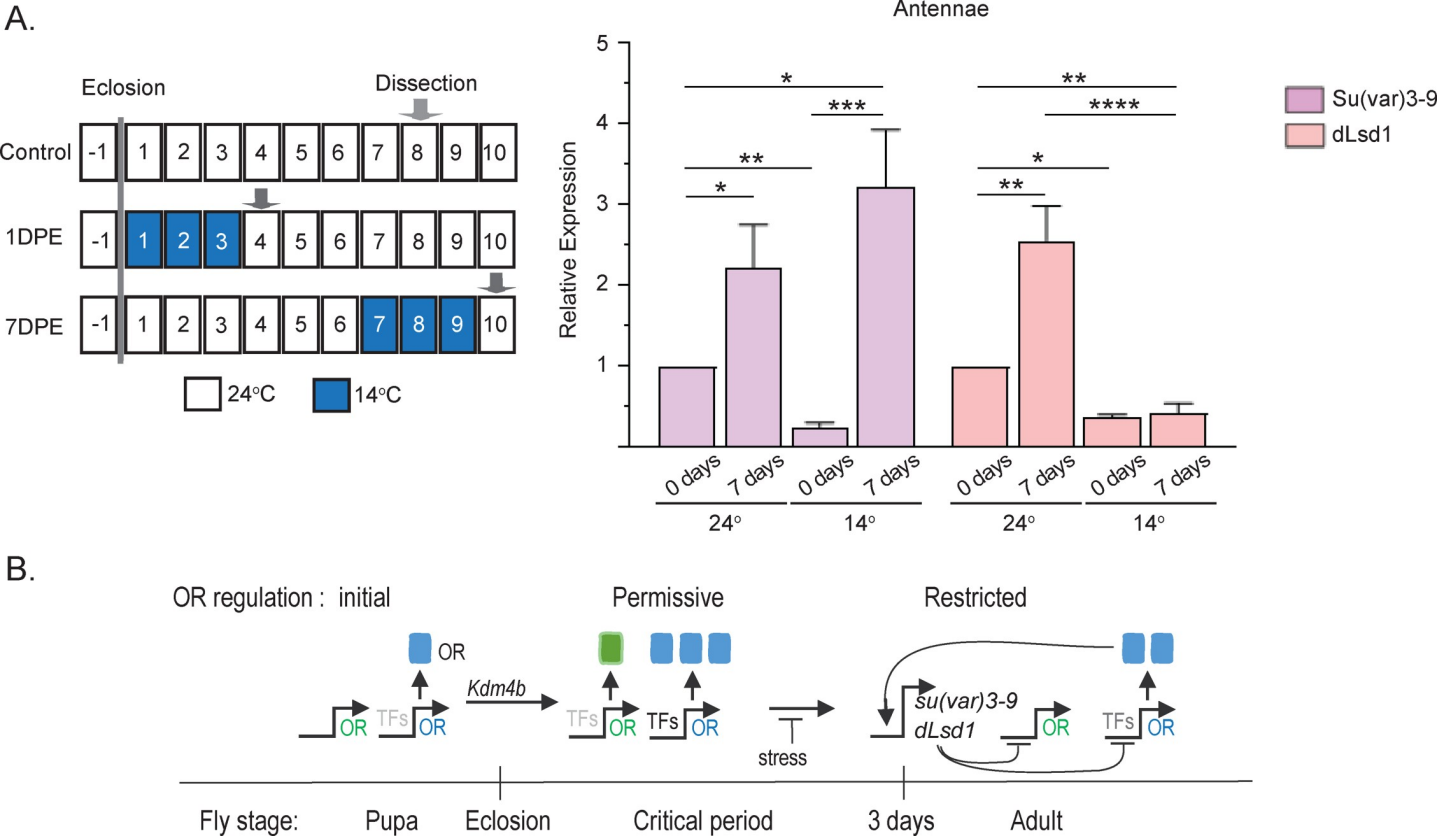
*dLsd1* regulation differs following environmental stress during or after the critical period. (A) Schematic showing the time points of thermal stress treatment and sample preparation. The graph shows the *Su(var)3-9* and *dLsd1* mRNA levels in the antenna after 3 days of thermal stress (14°C) ending at 4 DPE or 10 DPE, compared to flies maintained at ambient temperature (24°C) (S4 Data). (B) Schematic model of the progression of OR gene expression regulation from initiation to adult mature OR expression. OR proteins depicted as rectangles. DPE, days post-eclosion; OR, odorant receptor; TF, transcription factor.

To determine whether *dLsd1* and *Su(var)3-9* expression are sensitive to stress, we analyzed their expression in flies shifted to low temperature at different time points (Fig 7). Flies subjected to a temperature shift at eclosion (1 DPE) showed a 2-fold reduction in *dLsd1* and *Su(var)3-9* expression (Fig 7). The balanced reduction is consistent with a continuous permissiveness. Interestingly, reduction in copy number of both *Su(var)3-9* and *dLsd1* produced single-class expression, whereas stress produced ectopic expression, suggesting that additional stress signals enhance the permissive state. After a similar shift in adult flies (7 DPE), *dLsd1* expression fell to the level found at eclosion, whereas *Su(var)3-9* expression showed no significant change (Fig 7). The resulting imbalance between *Su(var)3-9* and *dLsd1* is consistent with the loss of *Or59b* expression we observed when we exposed adult heterozygous *dLsd1* mutant flies to stress (Fig 4C).

## Discussion

Here, we show that *Drosophila* OR expression matures and that OSNs become terminally differentiated after OR expression initiation. Our results show that OR expression matures in 3 steps: initiation, establishment, and refinement.

### OR expression initiation: Predetermined versus stochastic

Models of vertebrate OR expression suggest the existence of a heterochromatin switch that initiates expression [6,8]. We found *Kdm4b*, an H3K9me3 demethylase, induces OR expression. In a direct instructive model, a predetermined differentiation path produces TFs that recruit Kdm4b to open an OR locus. In a more stochastic model, Kdm4b opens chromatin at an OR locus, and if the necessary factors are available, the locus is kept open. A recent study revealed that low OR expression precedes the initiation step [41], which, together with our results, favors a model in which Kdm4b is recruited to the OR locus or even attracted by low OR expression, and in the presence of high OR expression inhibits OR initiation and expression at other OR loci.

### A deeply conserved OR maturation mechanism

The mechanism that establishes OR expression was first identified in mice [44]. Perhaps the most striking point of conservation is the unique OSN-specific function of *Lsd1*. In most *Drosophila* and vertebrate cells, Lsd1 erases H3K4 methylation and dimethylation and induces heterochromatin formation [36,37]. Our results and several vertebrate OR choice studies [6,45,46] show that Lsd1 opposes *Su(var)3-9* and constitutive heterochromatin formation in OSNs. The enzyme that forms H3K9me2, G9a, also restricts OR expression in both *Drosophila* and mice [24,47], making it clear that H3K9me2 lies at the center of OR gene regulation across phyla. The *cis* regulatory regions have also evolved to balance heterochromatin formation in similar ways between *Drosophila* and mice. TF cooperativity opposes heterochromatin formation and stabilizes *Or59b* expression [23]. In mice, *Lhx2*, one of the few TFs known to regulate vertebrate OR expression [34], also requires cooperativity to block heterochromatin formation and establish OR expression [35].

Also, the OR feedback loop was first described in mice [44,48]. The vertebrate feedback mechanisms build on the folding of [6,9] or signaling from [10,11] an expressed OR to inhibit the expression of other ORs. With the many levels of conserved features in OR regulation, it is possible that vertebrate-like OR feedback mechanisms link the expressed OR and *dLsd1* and *Su(var)3-9* expression.

After maturation, the OR expression mechanisms differ between *Drosophila* and mice. In *Drosophila*, both OR alleles are expressed in each OSN [49]. In mice, 1 OR allele is selected and expressed continuously by what is likely a separate mechanism. Another difference in *Drosophila* is that *dLsd1* activity balances *Su(var)3-9* activity after maturation, whereas in mice, Lsd1 is down-regulated after maturation [44]. We found that it is only *Lsd1* that is suppressed after maturation, suggesting that the memory mechanism that maintains strict monogenic OR expression is an inflexible *Su(var)3-9* expression that produces a defined heterochromatin level and sets the OR expression baseline. It remains unclear if such a memory mechanism is conserved, given the differences in regulation after OR expression maturation.

### A critical period mechanism controls OR expression

The restricted duration of OR expression maturation suggests that the period of gene regulation plasticity may be a bona fide critical period [50,51]. OR regulation does fulfill the criteria. First, a critical period should have a restricted duration, and OR expression maturation ends after a very sharp transition in gene regulation 2 DPE. Second, the plasticity of a critical period should be sensitive to activity in the circuit, and we found that feedback from an expressed OR can refine OR expression. Third, the phenotype changing in a critical period should be refined through competition, and we show that ectopic OR expression can outcompete endogenous OR expression. Fourth, the plasticity in a critical period should be sensitive to external stress, and we show that stress can dramatically alter OR expression during and after the relevant period. Fifth, the phenotype developing during a critical period becomes permanent after the period has passed, and we show that adult OR expression reaches a permanent state, indicating that OSN differentiation ends as the critical period closes. The conserved nature of the mechanisms, and the fact that immature vertebrate OSNs also show a low frequency of OR co-expression [52,53], suggests vertebrate OSN differentiation closes with a critical period as well.

### Differences in OR feedback regulation between OSN classes

We found that the timing of the OR feedback regulation depends on the OSN lineage. According to our results, most trichoid-related OR expression regulation changes take place in the pupal stage, whereas basiconic-related OR expression refinement seems to take place after eclosion. Interestingly, this difference in regulation relates to olfactory function because basiconic-related ORs respond more to food-related odorants while trichoid-related ORs respond more to pheromones for the sake of social interactions. When a fly emerges from its pupal case, it does so in the vicinity of the food it lived on as a larva but not necessarily close to other flies. Consistent with this, pheromone responses increase as social interactions increase post-maturation [54]. These response increases come, at least in part, from the sensitization of Or47b OSNs rather than from changes in *Or47b* expression, suggesting a separate mechanism. Thus, early trichoid-related OR gene regulation supports OR expression even in the absence of stimuli and allows for plasticity even after the OSNs have matured. For basiconic-related OR expression, the ORs’ late regulation provides more tuning possibilities in a dynamic food odor environment.

### The critical period provides flexibility for OR gene regulation

Predetermined systems of OR gene regulation lack the flexibility that feedback mechanisms can provide. We and others have shown that high odor responses suppress *Drosophila* OR expression [20,22]. Feedback mechanisms like this could tune responses to environmental odor levels and ensure odor responses fall within physiological limits. Our results further predict that feedback refinement buffers and allows for imperfect gene regulation, reducing the regulatory cost to maintain tight monogenic OR expression. The TFs required to express 1 particular OR can likely even vary with internal state and stress level. Our results also predict that the DNA binding motif locations and *cis* regulatory mechanisms can be plastic between species.

Stress inhibition of the feedback mechanisms also adds to the flexibility of the system. Stress can induce OR paralog expression, allowing the previously suppressed paralog to contribute to odor responses when the environment changes. In short-lived organisms like *Drosophila*, stress early in life predicts an insecure future. It therefore follows logically that stress early in life would inhibit OR expression maturation, and if the stress lasts beyond the critical period, the changes become permanent. In this way, the permissiveness built into the system makes OSNs and OR gene regulation more robust and resilient to continued or future episodes of stress.

The stress-altered OR expression also makes the animal more robust to environmental variability. Our results indicate that the feedback systems and the critical period function as a capacitator, silencing the effect of allelic variability, allowing changes in the OR genes and adaptation of olfactory function. This capacitator function hypothesis predicts that in the non-stressed ambient state, OR feedback keeps paralogs and alternate alleles dormant and produces the uniform OR expression observed in adult flies. But when the environment changes, stress blocks feedback suppression and dormant OR alleles or paralogs can be expressed, leading to an individualization of OR expression and odor responses in the population. Interestingly, the OSNs that express ORs also express IR co-receptors in both *Drosophila* and mosquitoes [55,56], suggesting that ORs and IRs are co-expressed in some OSN classes. Electrophysiology also shows that some OSN classes in *Drosophila* (ab1b, ab3a, and ab6a) respond to IR odors [57], suggesting that stress can tweak the balance between co-expressed ORs and IRs. Thus, our prediction is that stress accentuates alternative responses and OR allele expression when environmental conditions change, and shifts the system from optimal function to maximal detection.

## Materials and methods

### *Drosophila* stocks

The *Or59b* promoter fusion and *Or59b* minimal enhancer constructs were described previously [23]. *Pebbled-Gal4* (*Peb-Gal4*) was a kind gift from Liqun Luo (Stanford University, Stanford, CA, US). The *Su(var)3−9*^*06*^ and *Lsd1*^*09*^ mutants were a kind gift from Anita Öst (Linköping University, Linköping, Sweden). UAS-Or42b was a kind gift of Matthieu Louis, and UAS-Or47b:HA;UAS-Or65a was a kind gift from John Carlson. The following RNA interference (RNAi) lines were obtained from the Transgenic RNAi Project (TRiP; Harvard Medical School, Boston, MA, US; http://www.flyrnai.org): *Su(var)3-3 (dLsd1)-IR* (36867; 32853, 33726), *Kdm4a-IR* (34629), and *Kdm4b-IR* (35676, 57721). The following fly lines were provided by the Bloomington Drosophila Stock Center (Indiana University, Bloomington, IN, US; http://flystocks.bio.indiana.edu): *w*^*1118*^ (38690) and *Orco-Gal4* (23909).

### RNAi methodology and environmental experiments

Virgin RNAi females were mated with males carrying *Pebbled-Gal4*, *UAS-Dicer2*, and the cluster transgenes. The crosses were set up and maintained at 24°C. Then, 2–5 days after eclosion, the flies were dissected, stained, and scored for phenotypes.

For the stress experiments, flies were collected as virgins and raised on standard *Drosophila* culture medium at 24°C. On the day for the temperature shift, the temperature-stressed flies were transferred to new vials and maintained for 3 days at 14°C, while control flies were maintained at ambient temperature. Further information can be found in the supplemental experiment statics and details (S2 Data).

### Immunofluorescence

Immunofluorescence was performed as previously described [15]. The following primary antibodies were used: rabbit anti-GFP (1:2,000, TP-401; Torrey Pines Biolabs) and mouse anti-nc82 (1:100; Developmental Studies Hybridoma Bank). Secondary antibodies were conjugated with Alexa Fluor 488 (1:500; Molecular Probes) and Goat anti-Mouse IgG (H+L) Cross-Adsorbed Secondary Antibody, Rhodamine Red-X (1:250; Thermo Fisher Scientific). Confocal microscopy images were collected on an LSM 700 (Zeiss) and analyzed using the LSM Image Browser. The numbers of OSNs co-expressing BP104 and GFP for the different constructs were counted in these images. Adobe Photoshop CS4 (Adobe Systems) was used for image processing.

### Quantitative PCR

Antennae were obtained with a sieve after freezing the appropriate flies in liquid nitrogen. Total RNA from the antennae was extracted with TRIzol (Invitrogen) and purified with the RNeasy kit (Qiagen). Quantitative PCR was conducted on an Applied Biosystems 7900HT Fast Real-Time PCR System (Life Technologies) using the Power SYBR Green PCR Master Mix (Applied Biosystems, Life Technologies) and primer sets designed using Primer Express Software v3.0.1 (Integrated DNA Technologies). Actin 5c was used as an internal control. To amplify cDNA products and not genomic DNA, primers were designed to join the end of one exon with the beginning of the next exon. Quantitative PCR for each primer set was performed on both control and experimental samples for 40 cycles. Following amplification, melt curve analysis and ethidium bromide agarose gel electrophoresis were performed to evaluate the PCR products. The relative quantification of the fold change in mRNA expression was calculated using the 2−ΔΔCT threshold cycle method.

### Library preparation

For RNA-seq experiments, virgin flies were collected, and 50 antennae were handpicked, either immediately or after 4 or 14 days on standard *Drosophila* culture medium at 24°C. Total RNA was extracted using TRIzol (Invitrogen, cat. no. 15596–018) according to the manufacturer’s instructions. DNA was degraded using the Invitrogen TURBO DNA-*free* Kit. After DNase treatment, TRIzol RNA extraction was repeated a second time. The concentration and quality of the RNA was determined using a sensitive fluorescent-dye-based Qubit RNA HS Assay Kit and the Agilent HS RNA kit and an Agilent 4200 TapeStation System.

Using 1–5 μg of total RNA for each sample, we performed 2 rounds of mRNA isolation using the NEBNext Poly(A) mRNA Magnetic Isolation Module (E7490) according to the manufacturer’s instructions. Libraries were generated using the NEBNext RNA Ultra II RNA Library Prep Kit. The samples were quality controlled and successfully sequenced on an Illumina NextSeq 500 next-generation sequencing system in mid-output mode via 1 × 100 bp paired-end sequencing.

### RNA-seq analysis

The RNA read counts were estimated with Kallisto (version 0.45.1). Differentially expressed genes were estimated by DESeq2 (version 1.26.0) after counts had been rounded to the nearest integer count. The linear model was simply one group versus the other group, e.g., WT day 1 versus day 4, or WT day 1 versus treatment day 1. Plots were made using ggplot2 and R, showing log10 size-factor-normalized read counts.

## Supporting information

S1 Fig Difference between OSN lineages in when activity regulates OR expression. Degree of change in sequence counts observed between control and the different genotypes at 4 DPE relative to 1 DPE. Normalized logarithmic read counts (log10 size-factor-normalized) for each gene from the respective sample were scatter-plotted. Genes shown in grey except basiconic ORs (green) and trichoid ORs (magenta). The line is the reference at which gene expression is the same between conditions, with increased expression above, and suppression below, the line. Statistics for the figure are in S3 Data.
(TIF)

S1 Data The reads and statistics supporting Fig 1B–1E. (XLSX)

S2 Data The experimental outline and statistics for the *Or59b* marker experiments and quantitative PCR in Figs 2–5. (DOCX)

S3 Data The reads and statistics supporting Figs 3C–3E and S1. (XLSX)

S4 Data Raw results and statistics supporting Figs 6 and 7. (XLSX)

## Abbreviations

BBS: BBSome
DPE: days post-eclosion
EP: ethyl propionate
GR: gustatory receptor
H3K9me2: histone H3 lysine 9 dimethylation
H3K9me3: histone H3 lysine 9 trimethylation
IFT: intraflagellar transport
IR: ionotropic receptor
OBP: odorant binding protein
OR: odorant receptor
OSN: olfactory sensory neuron
RNA-seq: RNA sequencing
TF: transcription factor
