# Supplementary figures and images for: Stress and odorant receptor feedback during a critical period after hatching regulates olfactory sensory neuron differentiation in Drosophila
Source: PLoS Biol. 2021 Apr 1;19(4):e3001101. doi: 10.1371/journal.pbio.3001101 (PMC8043390; doi:10.1371/journal.pbio.3001101)

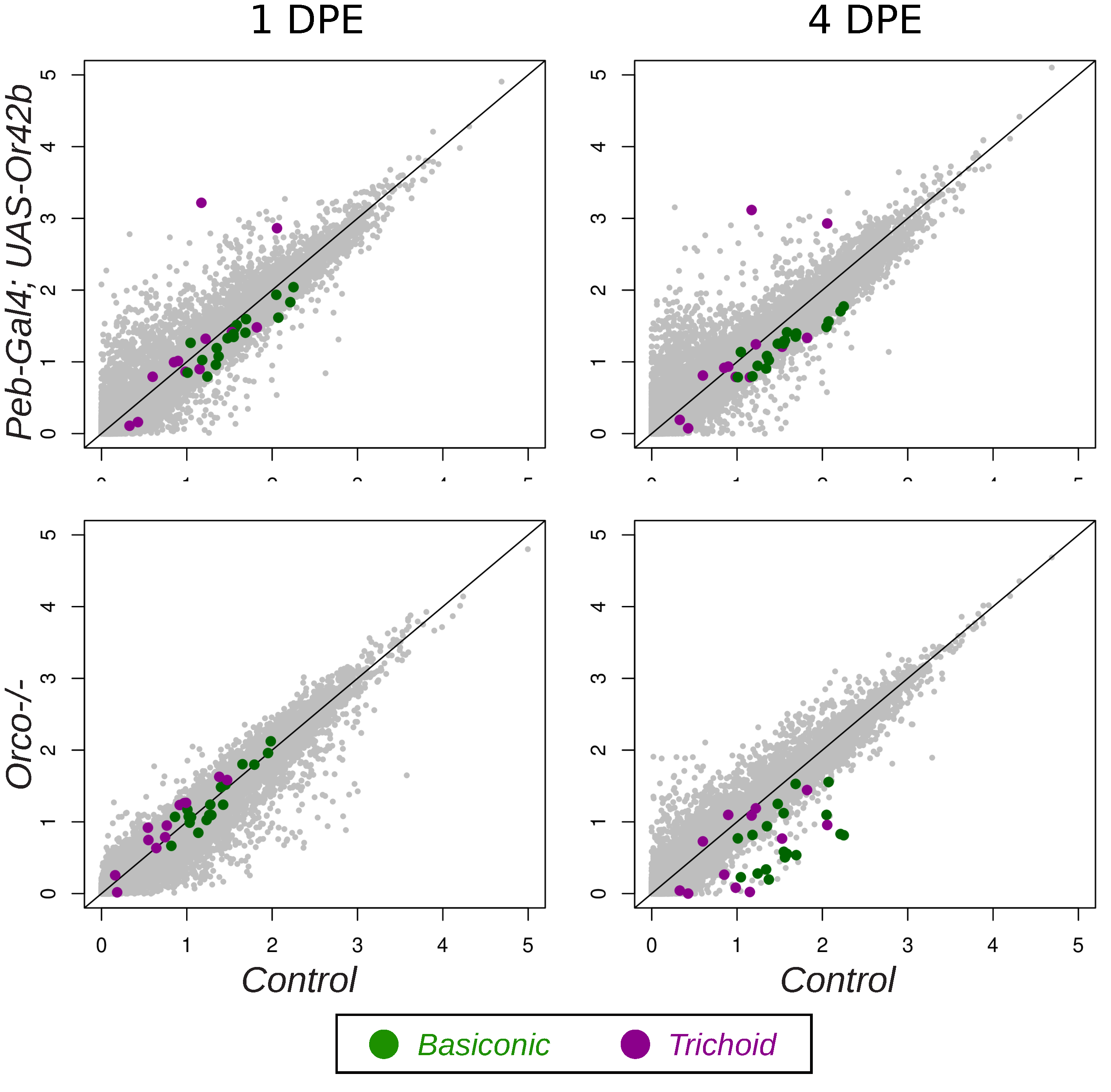

Supplement: S1 Fig — Degree of change in sequence counts observed between control and the different genotypes at 4 DPE relative to 1 DPE. Normalized logarithmic read counts (log10 size-factor-normalized) for each gene from the respective sample were scatter-plotted. Genes shown in grey except basiconic ORs (green) and trichoid ORs (magenta). The line is the reference at which gene expression is the same between conditions, with increased expression above, and suppression below, the line. Statistics for the figure are in S3 Data. (TIF) [file pbio.3001101.s001.tif]
