# Supplementary material for: Stress and odorant receptor feedback during a critical period after hatching regulates olfactory sensory neuron differentiation in Drosophila
Source: PLoS Biol. 2021 Apr 1;19(4):e3001101. doi: 10.1371/journal.pbio.3001101 (PMC8043390; doi:10.1371/journal.pbio.3001101)
Supplement: S2 Data — (DOCX) [file pbio.3001101.s003.docx]

**Experimental details and statistics:**

Figure 2A

Control. Virgins were collected and kept at 24⁰ for 7 days before dissection

Exp 1. Virgins were collected and kept at 14⁰ for 3 days before dissection

Exp 2. Virgins were collected and kept at 14⁰ for 3 days then moved back to 24⁰ for 7 more days before dissection

Exp 3. Virgins were collected and kept at 14⁰ for 3 days then moved back to 24⁰ for 18 more days before dissection

Exp 4. Virgins were collected and kept at 14⁰ for 3 days, moved back to 24⁰ for 3 days then moved back to 14⁰ for 3 days before dissection

***Or59bME* (10768-1-1 & 10768-1-9)***

| Tm Exp. |  | 14⁰ to 24⁰ |  |
| --- | --- | --- | --- |
|  | Lost (%) | Ectopic (%) | Control (%) |
| Control | 0 (0) | 0 (0) | 19 (100) |
| 1 | 0 (0) | 13 (65) | 7 (35) |
| 2 | 0 (0) | 14 (70) | 6 (30) |
| 3 | 0 (0) | 12 (55) | 10 (45) |
| 4 | 0 (0) | 10 (45) | 12 (55) |

*Reference flies were described at Jafari and Alenius, 2015.

Figure 2B

Control. Virgins were collected and kept at 24⁰ for 7 days before dissection

Exp 1. Virgins were collected and kept at 24⁰ for 3 days then moved to 14⁰ for 3 days before dissection

Exp 2. Virgins were collected and kept at 24⁰ for 3 days then moved to 14⁰ for 3 days then moved back to 24⁰ for 3 more days before dissection

Exp 3. Virgins were collected and kept at 24⁰ for 3 days then moved to 14⁰ for 3 days then moved back to 24⁰ for 14 more days before dissection

***Or59bME* (10768-1-1 & 10768-1-9)***

| Tm Exp. |  | 14⁰ to 24⁰ |  |
| --- | --- | --- | --- |
|  | Lost (%) | Ectopic (%) | Control (%) |
| Control | 0 (0) | 0 (0) | 20 (100) |
| 1 | 6 (32) | 2 (10) | 11 (58) |
| 2 | 6 (28) | 4 (19) | 11 (52) |
| 3 | 0 (0) | 1 (5) | 19 (95) |

*Reference flies were described at Jafari and Alenius, 2015.

Figure 3 A

***Or59b***

| Gene  Line | *Peb-Gal4* | | | *Peb-Gal4;UAS-Or47b* | | |
| --- | --- | --- | --- | --- | --- | --- |
|  | Lost | Ectopic | Wild type | Lost | Ectopic | Control |
| II | 0/20 | 0/20 | 20/20 | 10/20 | 0/20 | 10/20 |
| III | 0/20 | 0/20 | 20/20 | 8/18 | 0/18 | 10/18 |

Figure 3 B

***Or59b***

| Gene Line | *Peb-Gal4* | | | *Peb-Gal4;UAS-Or42b* | | | *Peb-Gal4;UAS-Or42b*+EP | | |
| --- | --- | --- | --- | --- | --- | --- | --- | --- | --- |
|  | Lost | Ectopic | Wild type | Lost | Ectopic | Wild type | Lost | Ectopic | Wild type |
| II | 0/20 | 0/20 | 20/20 | 1/18 | 0/18 | 17/18 | 4/21 | 0/21 | 17/21 |
| III | 0/20 | 0/20 | 20/20 | 3/18 | 0/18 | 15/18 | 4/22 | 0/22 | 18/22 |

Figure 4A

***Or59b***

| Gene Line | *Pbl-Gal4* (control) | | | *Peb-Gal4;UAS-Lsd1-RNAi* (B33726) | | | *Peb-Gal4;UAS-Lsd1-RNAi* (B36867) | | |
| --- | --- | --- | --- | --- | --- | --- | --- | --- | --- |
|  | Lost | Ectopic | Wild type | Lost | Ectopic | Wild type | Lost | Ectopic | Wild type |
| II | 0/20 | 0/20 | 20/20 | 1/9 | 0/9 | 8/9 | 2/10 | 0/10 | 8/10 |

| Gene Line | *Orco-Gal4* (control) | | | Orco-Gal4;UAS-Lsd1-IR (B33726) | | | Orco-Gal4;UAS-Lsd1-RNAi (B36867) | | |
| --- | --- | --- | --- | --- | --- | --- | --- | --- | --- |
|  | Lost | Ectopic | Wild type | Lost | Ectopic | Wild type | Lost | Ectopic | Wild type |
| II | 0/18 | 0/18 | 18/18 | 0/10 | 0/10 | 10/10 | 0/11 | 0/11 | 11/11 |

Figure 4B

***Or59bME***

| Gene Line | *Peb-Gal4 (control)* | | | *Peb-Gal4;UAS-Lsd1-RNAi* (B33726) | | | *Peb-Gal4;UAS-Lsd1-RNAi* (B36867) | | |
| --- | --- | --- | --- | --- | --- | --- | --- | --- | --- |
|  | Lost | Ectopic | Wild type | Lost | Ectopic | Wild type | Lost | Ectopic | Wild type |
| II | 0/15 | 0/15 | 15/15 | 5/9 | 0/9 | 3/9 | 2/7 | 0/7 | 5/7 |

| Gene Line | *Orco-Gal4* (control) | | | *Orco-Gal4;UAS-Lsd1-RNAi* (B33726) | | | *Orco-Gal4;UAS-Lsd1-RNAi* (B36867) | | |
| --- | --- | --- | --- | --- | --- | --- | --- | --- | --- |
|  | Lost | Ectopic | Wild type | Lost | Ectopic | Wild type | Lost | Ectopic | Wild type |
| II | 0/18 | 0/18 | 18/18 | 12/19 | 0/19 | 7/19 | 13/18 | 0/18 | 5/18 |

Figure 4C

***Or59bME***

| Gene Line | *su(var)3-3^09^ (dLsd1)* | | | *su(var)3-9^06^* | | | *su(var)3-3^09^/ su(var)3-9^06^* | | |
| --- | --- | --- | --- | --- | --- | --- | --- | --- | --- |
|  | Lost | Ectopic | Wild type | Lost | Ectopic | Wild type | Lost | Ectopic | Wild type |
| II | 12/20 | 0/20 | 8/20 | 7/22 | 4/22 | 11/22 | 0/19 | 0/19 | 19/19 |

Figure 5A

***Or59b***

| Gene Line | *Peb-Gal4* (control) | | | *Peb-Gal4;UAS-Kdm4a-RNAi* | | | *Peb-Gal4;UAS-Kdm4b-RNAi* | | |
| --- | --- | --- | --- | --- | --- | --- | --- | --- | --- |
|  | Lost | Ectopic | Wild type | Lost | Ectopic | Wild type | Lost | Ectopic | Wild type |
| II | 0/15 | 0/15 | 15/15 | 0/16 | 0/16 | 16/16 | 3/14 | 0/14 | 11/14 |

Figure 5B

***Or59b***

| Gene Line | *Orco-Gal4* (control) | | | *Orco-Gal4;UAS-Kdm4b-RNAi* | | |
| --- | --- | --- | --- | --- | --- | --- |
|  | Lost | Ectopic | Wild type | Lost | Ectopic | Wild type |
| II | 0/12 | 0/12 | 12/12 | 0/13 | 0/13 | 13/13 |

Figure 6E

***Or59b***

| Gene Line | *Peb-Gal4* (control) | | *su(var)3-9^06^* | | *Peb-Gal4;UAS-Or47b* | | *Peb-Gal4;UAS-Or47b/+; su(var)3-9^06^/+* | |
| --- | --- | --- | --- | --- | --- | --- | --- | --- |
|  | Lost | Wild type | Lost | Wild type | Lost | Wild type | Lost | Wild type |
| II | 0/30 | 15/30 | 0/23 | 23/23 | 15/31 | 16/31 | 0/29 | 29/29 |

Figure 7A

Control. Virgins were collected for RNA extraction

Exp 1. Virgins were collected and kept at 24⁰ for 7 days before RNA extraction

Exp 2. Virgins were collected and kept at 14⁰ for 3 days before RNA extraction

Exp 3. Virgins were collected and kept at 24⁰ for 6 days then moved to 14⁰ for 3 more days before RNA extraction
